# The underlying relationship between exercise and the prevalence of periodontitis: a systematic review and meta-analysis

**DOI:** 10.1186/s13102-023-00759-4

**Published:** 2023-11-27

**Authors:** Rongkai Cao, Piaopiao Qiu, Yuan Zhou, Bo Dong, Yucheng Han, Zhen Fan

**Affiliations:** Department of Implantology, Stomatological Hospital and Dental School of Tongji University, Shanghai Engineering Research Center of Tooth Restoration and Regeneration, No. 399, Middle Yanchang Rd, Shanghai, 200072 China

**Keywords:** Exercise, Periodontitis, Relationship, Systematic review

## Abstract

**Background:**

Although exercise has been proposed as an effective intervention for various lifestyle-related diseases and pathological scenarios, few researches assessed the impact of taking exercise on the prevalence of periodontitis.

**Purpose:**

This study aimed to perform a comprehensive literature review and meta-analysis from both observational and intervention studies to explore the relationship between exercise and periodontitis and to provide references for future intervention programs aimed at preventing periodontitis.

**Method:**

A systematic literature search was conducted in PubMed/MEDLINE, Web of Science, Cochrane Library, and Scopus for peer-reviewed studies published in English From January 1993 to January 2023 according to the PRISMA guidelines. Articles were selected if subjects were human and studies evaluating the association between exercise and periodontitis.

**Results:**

4098 references were retrieved. After screening the results, 30 studies were selected. Of these, 20 studies indicated an inverse association between exercise and periodontitis, while the remaining 10 did not reach conclusive findings. The meta-analysis demonstrated a risk ratio of 0.84 (95%CI: 0.77, 0.91) between the active group and the inactive group (P < 0.01), which indicated an inverse relationship between exercise and periodontitis.

**Conclusion:**

Overall, the increase in exercise presents an inverse association with the presence and severity of periodontitis. Accordingly, taking exercise might be a potential approach that contributes to improvements in periodontitis.

## Introduction

Exercise has been shown to have numerous beneficial effects on the general health of human beings and increase life expectancy [1]. Furthermore, studies have indicated the protective effects and improvement of taking moderate exercise on various lifestyle-related diseases including obesity, arthritis, hypertension, depression, and coronary heart disease [2]. Taking regularly moderate exercise can alleviate systemic inflammatory reactions and reduce the risk of multiple traditional chronic diseases [3]. Moreover, regular exercise has a positive impact on the management of stress and anxiety [4]. In addition to these physical and mental benefits, taking exercise may also help reduce healthcare costs and the need for medications through lower hospital stays and physician visits [5].

Periodontal health is defined as a state free from inflammation and characterized by the absence of gingival bleeding and shallow pockets [6]. Periodontitis is a chronic multifactorial inflammatory disease characterized by progressive destruction of the tooth-supporting apparatus and combined with dysbiotic plaque biofilms [7]. This disease is prevalent worldwide, affecting approximately 20–50% of the global population, making it a significant public health concern [8]. Although periodontitis mainly threatens oral health, its association with systemic conditions such as cardiovascular diseases, diabetes, and arthritis has also been established in the literature [9, 10]. However, given the high physical, psychological, social, and economic impacts on individuals and communities, prevention and management of periodontitis are crucial globally. A previous study summarized data related to severe periodontitis and indicated that governments should pay attention to the growing burden of severe periodontitis because of the increasing population associated with a better life expectancy around the world and a reduction of tooth loss [11].

Various lifestyle factors, including diet quality, alcohol consumption, and smoking, have been found to impact the occurrence and severity of periodontitis [8]. Substantial evidence suggests that interventions targeting lifestyle behaviors are effective, underscoring the importance of behavioral support in managing periodontitis [12]. Significant factors such as diet and smoking have been thoroughly investigated regarding their role in the risk of periodontal tissue decay [13, 14]. Nevertheless, there is limited research on the potential role of exercise in preventing periodontitis. Most studies regarded exercise as a part of lifestyle variables when evaluating the risk of periodontitis. However, few researchers investigated the level of exercise in detail, and the intervention studies are also limited in current literature.

Given that exercise has been shown to modulate systemic inflammatory reactions and alter inflammatory markers such as protein C-reactive levels, there may be a potential relationship between exercise and periodontitis [15, 16]. However, it is unclear whether exercise has a direct impact on periodontitis. Previous studies have tried to summarize the potential relationship between exercise and periodontitis [17, 18]. For example, a meta-analysis investigated the influence of physical exercise on periodontitis [17]. Another study also suggested an association between periodontitis and physical activity [18]. However, only 6 studies were included in this previous study [18]. Accordingly, the results should be considered carefully due to the limited research available and the certain risk of bias. Furthermore, only observational researches were included in these previous studies. Thus, the goal of this study was to perform a comprehensive meta-analysis from both observational and intervention studies to investigate the potential relationship between exercise and the prevalence of periodontitis and provide references for future intervention programs aimed at preventing periodontitis.

## Materials and methods

The present study was registered at PROSPERO under registration number CRD42023396334 and performed based on the Preferred Reporting Items for Systematic Reviews and Meta-analyses (PRISMA) guidelines [19]. Given that no human or animal subjects were involved in this study, medical ethics committee approval was not required.

The guiding question for this systematic review was formulated using the PICO format, with (P) indicating participants, (I) representing intervention, (C) standing for comparison, and (O) representing outcome [20]. Specifically, the guiding question was, “For individuals of all ages (P), what is the impact of exercise (I) or a sedentary lifestyle (C) on periodontitis (O)?” Participants of all ages were included. The intervention group contained subjects who engaged in regular or recommended exercise. The comparison group consisted of individuals who engaged in inadequate exercise or a sedentary lifestyle. The primary outcomes were the measurements of periodontitis.

### Search strategy

To identify relevant publications in accordance with PRISMA guidelines, a systematic literature search was performed in Web of Science, PubMed/MEDLINE, Scopus, and Cochrane Library databases. The search was limited to English-language publications published From January 1993 to January 2023. The search strategy used predefined search terms related to exercise and periodontitis, and the specific search strategies used for each database are provided in Table 1. Gray literature searches were also conducted on the International Clinical Trials Registry Platform of the World Health Organization and SciELO. To complement the study, the search in the databases was combined with a manual retrieval of the reference lists from the selected studies.

**Table 1:** Electronic databases used and search strategies

Database	Search strategy
PubMed	(“exercise“[MeSH Terms] OR “exercise“[All Fields] OR “exercises“[All Fields] OR “exercise therapy“[MeSH Terms] OR (“exercise“[All Fields] AND “therapy“[All Fields]) OR “exercise therapy“[All Fields] OR “exercise s“[All Fields] OR “exercised“[All Fields] OR “exerciser“[All Fields] OR “exercisers“[All Fields] OR “exercising“[All Fields] OR (“physical fitness“[MeSH Terms] OR (“physical“[All Fields] AND “fitness“[All Fields]) OR “physical fitness“[All Fields]) OR (“exercise“[MeSH Terms] OR “exercise“[All Fields] OR (“physical“[All Fields] AND “activity“[All Fields]) OR “physical activity“[All Fields])) AND (“periodontal diseases“[MeSH Terms] OR (“periodontal“[All Fields] AND “diseases“[All Fields]) OR “periodontal diseases“[All Fields] OR (“periodontal“[All Fields] AND “disease“[All Fields]) OR “periodontal disease“[All Fields] OR (“periodontal“[All Fields] OR “periodontally“[All Fields] OR “periodontically“[All Fields] OR “periodontics“[MeSH Terms] OR “periodontics“[All Fields] OR “periodontic“[All Fields] OR “periodontitis“[MeSH Terms] OR “periodontitis“[All Fields] OR “periodontitides“[All Fields] OR (“gingiva“[MeSH Terms] OR “gingiva“[All Fields] OR “gingival“[All Fields] OR “gingivally“[All Fields] OR “gingivals“[All Fields] OR “gingivitis“[MeSH Terms] OR “gingivitis“[All Fields] OR “gingivitides“[All Fields])))
Scopus	(TITLE-ABS-KEY (exercise) OR TITLE-ABS-KEY (exercises) OR TITLE-ABS-KEY (exercise therapy) OR TITLE-ABS-KEY (exercise’s) OR TITLE-ABS-KEY (exercised) OR TITLE-ABS-KEY (exerciser) OR TITLE-ABS-KEY (exercisers) OR TITLE-ABS-KEY (exercising) OR TITLE-ABS-KEY (physical fitness) OR TITLE-ABS-KEY (physical activity)) AND (TITLE-ABS-KEY (periodontal diseases) OR TITLE-ABS-KEY (periodontal disease) OR TITLE-ABS-KEY (gingiva) OR TITLE-ABS-KEY (gingival) OR TITLE-ABS-KEY (gingivally) OR TITLE-ABS-KEY (gingivals) OR TITLE-ABS-KEY (gingivitis) OR TITLE-ABS-KEY (gingivitides))
Web of science	TS= (exercise OR exercises OR exercise therapy OR exercise s OR exercised OR exerciser OR exercisers OR exercising OR physical fitness OR physical activity) AND TS= (periodontal diseases OR periodontal disease OR periodontal OR periodontally OR periodontically OR periodontics OR periodontic OR periodontics OR periodontitides OR gingiva OR gingival OR gingivally OR gingivals OR gingivitis OR gingivitides)
Cochrane library	(exercise OR exercises OR exercise therapy OR exercise s OR exercised OR exerciser OR exercisers OR exercising OR physical fitness OR physical activity) AND (periodontal diseases OR periodontal disease OR periodontal OR periodontally OR periodontically OR periodontics OR periodontic OR periodontics OR periodontitides OR gingiva OR gingival OR gingivally OR gingivals OR gingivitis OR gingivitides)

### Eligibility criteria

The following criteria were applied to choose publications: (1) peer-reviewed research articles published in English, (2) studies conducted on human subjects with no age restrictions, and (3) observational or experimental studies that examined the relationship between exercise and periodontitis.

The following exclusion criteria were performed: (1) studies conducted on animals, case reports, letters, editorials, conference abstracts, and review articles; (2) studies with insufficient data to extract relevant information including the exercise measurement and evaluation of periodontitis; and (3) publications written in languages other than English.

### Study selection

The information obtained by search strategy in each database was collated, and duplicate entries were eliminated. Two reviewers independently assessed the abstract and title of the retrieved articles based on eligibility criteria. Studies which were considered ineligible by both reviewers were promptly excluded, while studies deemed ineligible by one author but eligible by another were retained for full-text evaluation. The reviewers worked in tandem to analyze articles not excluded in their entirety. Publications that satisfied the eligibility criteria were used for further data extraction. Any discrepancies were resolved by means of discussion.

Data from the included studies were retrieved in detail. Report of the following variables was extracted: author(s), year of publication, study type, country, number of participants, gender, age, exercise assessment, evaluation of periodontitis, main findings, and conclusions.

### Quality assessment

The risk of bias for observational studies was evaluated in accordance with the “Quality Assessment Tool for Observational Cohort and Cross-Sectional Studies” and the “Quality Assessment of Case-Control Studies” from the National Institutes of Health (Available online at https://www.nhlbi.nih.gov/health-topics/study-quality-assessment-tools). These tools comprise 14 criteria (12 for case-control studies) designed to appraise the internal validity of cross-sectional studies, case-control studies, and cohort studies. Responses to each criterion could be “yes,” “no,” “not applicable,” or “not reported.” Responses other than “yes” indicate a potential risk of bias. After evaluation, articles were defined as good, fair, or poor. Studies rated as “good” had a maximum of three domains that were not answered as “no” or “not applicable.” The domains of “Validity of outcomes” and “Adjustment for confounders” were considered the most critical factors in determining the classification of poor study quality.

Furthermore, the risk of bias for experimental researches was assessed based on the Cochrane Collaboration’s tool, using Review Manager 5.4 (The Nordic Cochrane Center, The Cochrane Collaboration, Copenhagen, Denmark) [21]. The tool contains the following criteria: allocation concealment, random sequence generation, blinding of outcome assessment, blinding of participants, incomplete outcome data, blinding of operator, and selective reporting. Each study’s risk of bias was classified as low, with some concerns, or high based on these criteria.

The same reviewers independently analyzed the included studies using the above tools. In case of any discrepancies, a consensus decision was made through further discussion with a third review author.

### Data synthesis

Data on exercise and periodontitis were extracted and analyzed using Stata 17.0 Software (College Station, TX: StataCorp LLC, USA) to evaluate the underlying relationship between exercise and periodontitis. Specifically, the prevalence of periodontitis and the number of individuals in active and inactive groups were recorded to determine the odds ratio with a 95% confidence interval (95%CI). Heterogeneity was calculated using the I^2^ test, which measures the rate of variation among articles caused by heterogeneity instead of probabilistic chance. The fixed-effects model and the random-effects model were used to test the significance of treatment effects in case of absence and high heterogeneity, respectively [22]. The estimated effect was regarded as significant where *P* < 0.05.

## Results

### Study selection

**Fig. 1:**
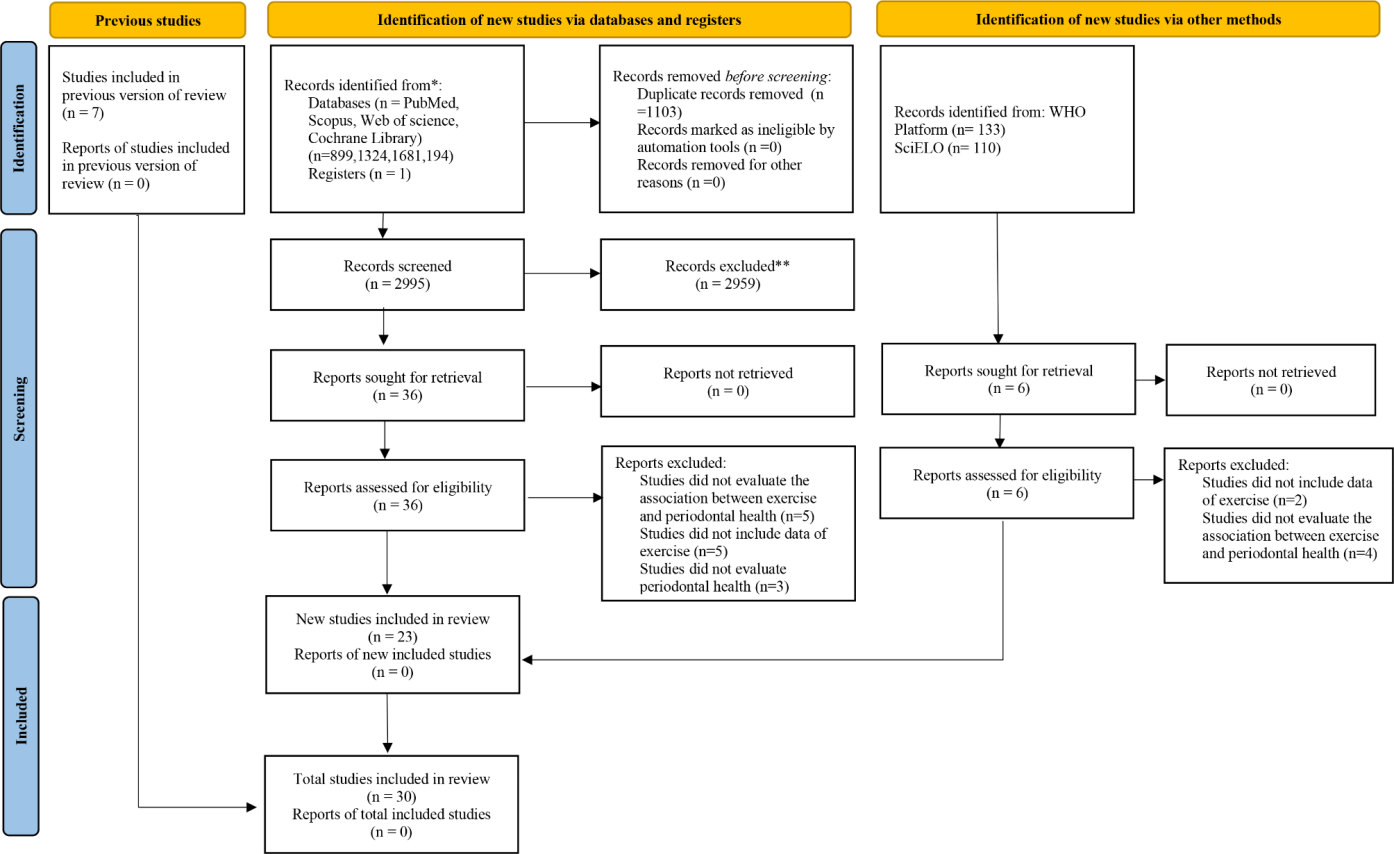
Flow chart of the literature search and results

4,098 papers were initially obtained through an electronic search, comprising 899 articles from PubMed/MEDLINE, 1,324 articles from Scopus, 1,681 articles from Web of Science, and 194 articles from Cochrane Library. Furthermore, a manual search of manufacturers’ reference lists yielded seven additional articles. None of the 243 references from gray literature was considered eligible. 2,995 studies remained after removing duplicates, and 2959 of them were excluded after assessing the titles and abstracts. After thoroughly examining the full texts, 13 studies were excluded since they did not meet the eligibility criteria. At last, 30 articles were selected for inclusion in this systematic review (Fig. 1).

### Characteristics of the studies

Tables 2, 3 and 4 provide detailed information on the 30 studies included in this review, of which 4 were experimental studies and 26 were observational studies. 20 of the included articles were cross-sectional design and most of them were performed in developed countries. Subjects of all ages were included, with a minimum of 25 and a maximum of 16,726. There was considerable variation in the tools used to assess physical activity, including different versions of questionnaires, strength, and maximal oxygen consumption. 18 studies elaborated on the types of sports, and 14 studies reported the duration time of measurement. The frequency and intensity of exercise have also been included in most selected studies. The assessment of periodontitis included clinical attachment loss and periodontal probing depth in most studies, with additional measures including gingival index, plaque index, bleeding on probing, and community periodontal index. The selected studies also investigated sedentary behavior, diet quality, normal body weight, alcohol consumption, and cigarette smoking as potential co-factors. Overall, from the 30 included studies, 20 studies reported that taking exercise was combined with a low prevalence of periodontitis, while the remaining 10 studies did not reach conclusive findings. For example, a representative cross-sectional study indicated that a higher prevalence of periodontitis was found in those inactive individuals compared to partially active individuals and subjects took recommended exercise [25]. A low level of exercise combined with a poor diet demonstrated a significant association with increased odds of periodontitis in another included study [27]. Possible explanations can be obtained from the included intervention studies. One research concluded that taking exercise may alter total salivary antioxidants activity, which could eventually affect periodontal health status [44]. Another study indicated that leisure-time exercise may protect against an excessive inflammatory response in periodontitis [46]. In addition, none of the selected studies reported a negative effect. Other main potential co-factors reported in the selected studies include maintaining a normal weight, consuming a healthy diet, sedentary behavior, and cigarette smoking.

**Table 2:** Characteristics of the included studies

Author(s)	Type of study	Country	Participants	Age	Evaluation of periodontal health
Alkan et al. [23] 2020	Before-after	Turkey	25 females	Periodontitis: 49.1(7.9); Health: 45.9(8.3)	PI, GI, PDD, BOP, GR and CAL
Almohamad et al. [24] 2022	Cross-sectional	USA	1643 males and 1684 females	> 30	CAL and PDD
Al-Zahrani et al. [25] 2005	Cross-sectional	USA	1245 males and 1276 females	Periodontitis: 50.3(0.9); Health: 47.4(0.5)	CAL and PDD
Al-Zahrani et al. [26] 2005	Cross-sectional	USA	12,110	> 18	CAL and PDD
Bawadi et al. [27] 2011	Cross-sectional	Jordan	168 males and 172 females	36(14.9)	PI, GI, CAL and PDD
Bazyar et al. [28] 2019	Case control	Iran	67 males and 136 females	Case: 51.1(5.7) Control: 50.8(5.4)	BOP, PI, PDD and CAL
Cueto et al. [29] 2005	Case control	Spanish	89 males and 60 females	Case: 62.5(9.9) Control: 58.5(10.2)	PDD, CAL
Han et al. [30] 2010	Cross-Sectional	Korean	457 males and 589 females	42.3(12.2)	CPI
Han et al. [31] 2016	Cross-Sectional	Korean	16,726 males	Case: 52.08(0.31) Control:40.57(0.26)	CPI
Han et al. [32] 2017	Cross-Sectional	Korean	14,527 males	Case: 54.1(0.3) Control: 41.8(0.2)	CPI
Han et al. [33] 2018	Cross-Sectional	Korean	6117 males and 8558 females	> 19	CPI
Han et al. [34] 2019	Cross-sectional	Korea	5175 males and 6746 females	45.9	CPI
Hasan et al. [35] 2021	Case Cohort	Bangladesh	175 males and 204 females	21–60	CPI
Hoppe et al. [36] 2017	Cross-Sectional	Brazil	112 males	34.9(10.3)	PD, AL
Hwang et al. [37] 2022	Cross-sectional	Korea	5545 males and 7144 females	> 19	CPI
Iwasaki et al. [38] 2023	Cross-sectional	Japan	746 males and 1414 females	58.1(9.6)	CAL, PDD and GR
Kongstad et al. [39] 2017	Cross-Sectional	Denmark	1425 males and 2240 females	53.7(13.7)	CAL, PDD and GR
Marruganti et al. [40] 2022	Cross-sectional	Italy	99 males and 136 females	53.9	clinical attachment level, Probing depth, gingival recession (REC), plaque and Bleeding on Probing
Marruganti et al. [41] 2023	Cross-sectional	USA	5264 males and 5415 females	50.8(0.3)	PDD and GR
Mendoza-Núñez et al. [42] 2014	Intervention	Mexico	71	60–74	PDI
Merchant et al. [43] 2003	Case Cohort	USA	2123 males	40–75	Radiographic bone loss, viewing box and magnifying loupes
Munther et al. [44] 2019	Case Cohort	Iraq	120 males	20–25	Periodontal Disease Index
Sakki et al. [45] 1995	Cross-sectional	Finland	226 males and 261 females	55	PDD
Sanders et al. [46] 2009	Case control	Australia	310 males and 440 females	> 18	PDD and GR
Samnieng et al. [47] 2013	Cross-sectional	Thailand	158 males and 454 females	68.8(5.9)	CAL and PDD
Schmidt et al. [48] 2022	Cross-sectional	Germany	568 males and 590 females	13.2(2.3)	CPI
Oliveira et al. [49] 2015	Cross-sectional	Brazil	111 males	34.8(10.3)	BOP, CAL and PDD
Omori et al. [50] 2018	Intervention	Japan	71 males	31–64	PPD, BOP
Wernicke et al. [51] 2021	RCT	Germany	37	> 18	PPD, BOP and plaque scores
Yu et al. [52] 2011	Cross-Sectional	USA	1902 males and 1954 females	71.2(7.7)	CAL and PDD

CAL: clinical attachment loss; PDD: periodontal probing depth; PI: plaque index; GI: gingival index; BOP: bleeding on probing; GR: gingival recession; CPI: community periodontal index; GCF: gingival crevicular fluid; PDI: Periodontal Disease Index;

**Table 3:** Details of exercise in the included studies

Author(s)	Exercise measurement	Type of exercise	Duration time	Frequency	Intensity of exercise
Alkan et al. [23] 2020	Maximal oxygen consumption, handgrip and backleg strength	N/A	12 weeks	One hour per day, 3 days a week	N/A
Almohamad et al. [24] 2022	IPAQ	N/A	N/A	N/A	Moderate or high
Al-Zahrani et al. [25] 2005	Questionnaires on frequency of nine leisure-time physical activities	Walking a mile or more at a time without stopping, jogging or running, bike riding, aerobics exercise, dancing, swimming, calisthenics, garden or yard work and weight lifting	10 years or longer	Moderately intense physical activity per week or three episodes of vigorously intense activity per week	Moderate or vigorous
Al-Zahrani et al. [26] 2005	Questionnaires on frequency of nine leisure-time physical activities	Walking a mile or more at a time without stopping, jogging or running, bike riding, aerobic dancing or exercise, dancing, swimming, calisthenics, garden or yard work, and weight lifting	N/A	≥ 5 episodes of moderate or ≥ 3 episodes of vigorous-intensity physical activity per week	Moderate or vigorous
Bawadi et al. [27] 2011	IPAQ	Heavy lifting, digging, aerobics, or fast bicycling	7 days	N/A	Low or high
Bazyar et al. [28] 2019	IPAQ	N/A	N/A	N/A	N/A
Cueto et al. [29] 2005	Regular practice of physical exercise	N/A	N/A	Regular	N/A
Han et al. [30] 2010	Physical activity in a week	Walking or exercise	1 week	N/A	N/A
Han et al. [31] 2016	Regular exercisers if they performed walking at least 5 times per week for over 30 min per session	N/A	N/A	N/A	N/A
Han et al. [32] 2017	Regular exercisers if they performed walking at least 5 times per week for over 30 min per session	N/A	N/A	Regular	N/A
Han et al. [33] 2018	Regular exercisers if they performed walking at least 5 times per week for over 30 min per session	Walking	N/A	Five times per week for over 30 min per session	N/A
Han et al. [34] 2019	IPAQ	Walking	1 week	Walking for > 30 min, ≥5times a week	N/A
Hasan et al. [35] 2021	IPAQ	Moderate physical activity, vigorous physical activity	7 days	[moderate physical activity + vigorous physical activity × 2] ≥ 150 min in 7 days	Moderate or vigorous
Hoppe et al. [36] 2017	The number of days per week that the individual engaged in physical exercise	Running	Not exceeding 3 months	2 times per week	N/A
Hwang et al. [37] 2022	IPAQ	Moderate-intensity activity and high-intensity activity	N/A	2.5 h or more of moderate-intensity physical activity, 1.25 h of high-intensity physical activity, or a combination of moderate-intensity and high-intensity physical activity per week	Moderate or high
Iwasaki et al. [38] 2023	The number of hours spent engaging in activities with different intensity levels in nonleisure time on a typical day in the last year and the frequency and number of hours spent engaging in activities with different intensity levels during leisure time	Walking slowly, walking quickly, light-to-moderate exercise, and strenuous exercise	1 year	N/A	Light, moderate and strenuous
Kongstad et al. [39] 2017	Leisure time physical activity was dichotomized as less than 4 h a week and ≥ 4 h a week	N/A	1 week	less than 4 h a week and ≥ 4 h a week	N/A
Marruganti et al. [40] 2022	IPAQ	N/A	7 days	N/A	Moderate, vigorous
Marruganti et al. [41] 2023	IPAQ	Moderate and vigorous intensity physical activity	1 week	N/A	Moderate, vigorous
Mendoza-Núñez et al. [42] 2014	Performed Tai Chi 5 days a week for 60-minute sessions	Tai Chi	6 months	5 days a week	N/A
Merchant et al. [43] 2003	h per week on average spent in the last year walking, hiking, jogging, running, bicycling, swimming, playing tennis, squash, or aerobics, and the number of stairs climbed.	Walking (including walking to work), hiking, jogging, running, bicycling, using a stationary bicycle, swimming, playing tennis, squash, or aerobics, and the number of flights of stairs they climbed	N/A	N/A	Light, moderate, strenuous
Munther et al. [44] 2019	Physical exercises were performed for a half-hour to an hour daily either at home or the gym	N/A	N/A	a half-hour to an hour daily	N/A
Sakki et al. [45] 1995	Low if subject used less than 15 min for walking or driving a bike on the way to work and exercised only once or less in a week during leisure time. Otherwise it was rated as high.	Walking or driving a bike	N/A	Physical activity was low if the subject used less than 15 min for walking or driving a bike on the way to work and exercised only once or less in a week during leisure time. Otherwise it was rated as high.	N/A
Sanders et al. [46] 2009	Eight core questions from the Active Australia Survey	Continuous walking for at least 10 min.; vigorous gardening or heavy work around the yard; other vigorous activity; and other more moderate physical activities	N/A	at least five sessions of physical activity per week culminating in at least 2.5 h of activity	Light, moderate, strenuous
Samnieng et al. [47] 2013	The number of desirable health practices.	N/A	N/A	Regular	N/A
Schmidt et al. [48] 2022	Asking the children whether and how often they engage (a) in organized physical and (b) non-organized physical activity	N/A	N/A	at least once a week, less than once a week	N/A
Oliveira et al. [49] 2015	PFT	Push-ups, sit-ups and running for 12 min.	N/A	N/A	N/A
Omori et al. [50] 2018	Resistance training or aerobic training in cycling session for 20 ~ 40 min at 60–85% maximal oxygen consumption	Resistance training or aerobic training in cycling session for 20 ~ 40 min	1 month	N/A	Moderate, vigorous
Wernicke et al. [51] 2021	Strength endurance and endurance	Aerobic exercise, resistance training, combined aerobic and resistance training	26 weeks	twice a week	Light, moderate, strenuous
Yu et al. [52] 2011	Activities of daily living, instrumental activities of daily living; leisure and social activities, lower extremity mobility, and general physical activities.	Stooping, crouching, kneeling, standing for 2 h, sitting for 2 h, standing up from an armless chair, reaching overhead, grasping and holding small objects, and lifting or carrying 10 pounds	N/A	N/A	Light, moderate, strenuous

IPAQ, International Physical Activity Questionnaire; PFT: physical fitness test;

**Table 4:** Main findings of the included studies

Author(s)	Main findings	Conclusions
Alkan et al. [23] 2020	A significant decrease in PB, CAL, serum leptin, GCF tumor necrosis factorα and leptin, and a significant increase in GCF resistin were observed in the chronic periodontitis.	Regular exercise exerts different impacts with respect to clinical and biochemical aspects of periodontal and systemic conditions in obese women.
Almohamad et al. [24] 2022	Individuals with higher total physical activity, higher leisure time physical activity, and lower amount of total sedentary behaviour had lower periodontal disease prevalence.	Higher sedentary behaviour is associated with higher odds of periodontal disease.
Al-Zahrani et al. [25] 2005	Prevalence of periodontitis was higher among inactive individuals than partially active individuals and those who met the recommended level of exercise.	Engaging in the recommended level of exercise is associated with lower periodontitis prevalence, especially among never and former smokers.
Al-Zahrani et al. [26] 2005	Individuals who maintained normal weight, engaged in the recommended level of exercise, and had a high-quality diet were 40% less likely to have periodontitis compared to individuals who maintained none of these health-enhancing behaviors.	An increased number of health-enhancing behaviors is associated with a lower periodontitis prevalence.
Bawadi et al. [27] 2011	Individuals who were highly physically active had a significantly lower average PI, GI, CAL and percentage of sites with CAL > 3 mm compared to individuals with a low level of physical activity and individuals with a moderate level of physical activity.	A low physical activity level and a poor diet were significantly associated with increased odds of periodontal disease.
Bazyar et al. [28] 2019	The results of the study showed that there is a significant inverse correlation between physical activity and weight, BMI, BOP, PI, PDD and CAL.	Reduction of metabolic and anthropometric parameters can improve periodontal status in T2DM patients with PD.
Cueto et al. [29] 2005	No association was found between regular practice of physical exercise and periodontitis.	There is evidence of an association between periodontitis and acute myocardial infarction.
Han et al. [30] 2010	An underlying relationship was found between physical activity in a week and periodontitis.	Metabolic syndrome might be associated with periodontitis.
Han et al. [31] 2016	There was associations between exercise and periodontal disease and severe periodontitis	Excessive consumption of green tea may be considered as a risk factor for periodontal disease.
Han et al. [32] 2017	An underlying relationship was found between regular exercise and the presence of periodontal disease.	The association between oral health behavior and periodontitis was proven.
Han et al. [33] 2018	Exercise was associated with periodontitis in men. By contrast, exercise was not associated with periodontitis in women.	Long sleep duration was associated with periodontitis.
Han et al. [34] 2019	In all models, subjects who walked regularly had signifcantly lower risks of periodontitis.	Regular walking is associated to lower prevalence of periodontitis.
Hasan et al. [35] 2021	The odds of periodontal disease increased with unfavourable glycaemic control, and decreased by 85% with adherence to physical activity.	Self-care practices, and oral hygiene practices must be taken into consideration for prevention of periodontal disease in patients with diabetes.
Hoppe et al. [36] 2017	No association was found between regular exercise and periodontitis.	The results suggest that oral inflammatory burden was associated with physical fitness.
Hwang et al. [37] 2022	Each of the HLS (diet quality, physical activity, normal body weight) practices was significantly associated with periodontal diseases.	In addition to improving oral hygiene, improving HLS should be emphasized for patients with periodontal diseases.
Iwasaki et al. [38] 2023	Total physical activity was inversely associated with the presence and severity of periodontitis in women. By contrast, physical activity was not associated with periodontitis in men.	Total physical activity had an inverse, linear association with the presence and severity of periodontitis in Japanese women but not in Japanese men.
Kongstad et al. [39] 2017	There was no statistically significant relationship of the lifestyle factors, alcohol consumption, diabetes, physical inactivity, BMI, WC, body fat, triglycerides, total cholesterol, or CRP and periodontitis	Regression analyses showed little difference in OR across the five periodontitis case definitions, however, the level of significance did show some variation.
Marruganti et al. [40] 2022	A high physical activity was significantly associated with a lower prevalence of Stage III/IV periodontitis compared to low/moderate physical activity.	Individuals conducting a lifestyle characterized by lack of regular exercise had 10 times the odds to have severe forms of periodontitis.
Marruganti et al. [41] 2023	High leisure-time physical activity as protective indicator for periodontitis, while high Occupational Physical Activity resulted as a significant risk indicator.	Leisure-time and occupational physical activity demonstrated divergent associations with periodontitis.
Mendoza-Núñez et al. [42] 2014	A statistically significant decrease in the PDI was observed in subjects who performed Tai Chi during a period of 6 months.	Practice of Tai Chi has both antioxidant and anti-inflammatory effects that are linked to the improvement of PD in older adults.
Merchant et al. [43] 2003	Compared to men in the lowest quintile of physical activity, those in the highest quintile had a 13% lower risk of periodontitis.	An inverse, linear association between sustained physical activity and periodontitis independent of known risk factors.
Munther et al. [44] 2019	The mean of PI was significantly higher among those who did not exercise compared to those who exercised. Smoking and physical exercises recorded a significant effect on the mean of the PI.	Physical exercise may alter total salivary antioxidants activity and the periodontal health status.
Sakki et al. [45] 1995	Lifestyle had an independent association with periodontal health. Periodontal pocketing increased with an unhealthier lifestyle.	Lifestyle could explain some of the social and sex differences in periodontal health.
Sanders et al. [46] 2009	Those meeting a prescribed threshold for leisure-time physical activity had lower adjusted odds of elevated IL-1b and detectable CRP than less active adults.	Leisure-time physical activity may protect against an excessive inflammatory response in periodontitis.
Samnieng et al. [47] 2013	Subjects who had no regular physical activity had a significantly higher prevalence of periodontal disease and lower salivary flow rate than their counterpart.	Good health practices were related with good oral health behaviors. Improving general health habits are suggested to lead to better oral health for the elderly, and vice versa.
Schmidt et al. [48] 2022	Physical activity in a sports club was associated with lower caries experience and periodontal health score.	Physical activity and high socioeconomic status are potentially protective with unfavorable oral health conditions.
Oliveira et al. [49] 2015	A 1 mm increment in PDD or CAL significantly decreased the chance of reaching the highest PFT score by 69% or 75%, respectively. Individuals presenting at least one tooth with AL ≥ 4 mm had significantly lower PFT scores compared with those without this status.	Periodontal disease may be considered a risk indicator for poor physical fitness in males.
Omori et al. [50] 2018	In the exercise intervention group, the number of teeth with a PPD ≥ 4 mm significantly decreased from 14.4–5.6%, and the number of teeth with BOP significantly decreased from 39.8–14.4%.	Exercise might contribute to improvements in periodontal disease.
Wernicke et al. [51] 2021	Both the BOP and the severity of periodontitis were significantly reduced in the intervention group compared to the control group.	Physical activity over a period of 6 months is a health promoting measure for patients with T2DM and improves periodontal health.
Yu et al. [52] 2011	The trends toward disability in general physical activities were statistically significant across increasing severity of oral health problems.	Poor oral health, specifically edentulism and severe periodontitis, is associated with multiple domains of late-life disability.

### Risk of bias

**Fig. 2:**
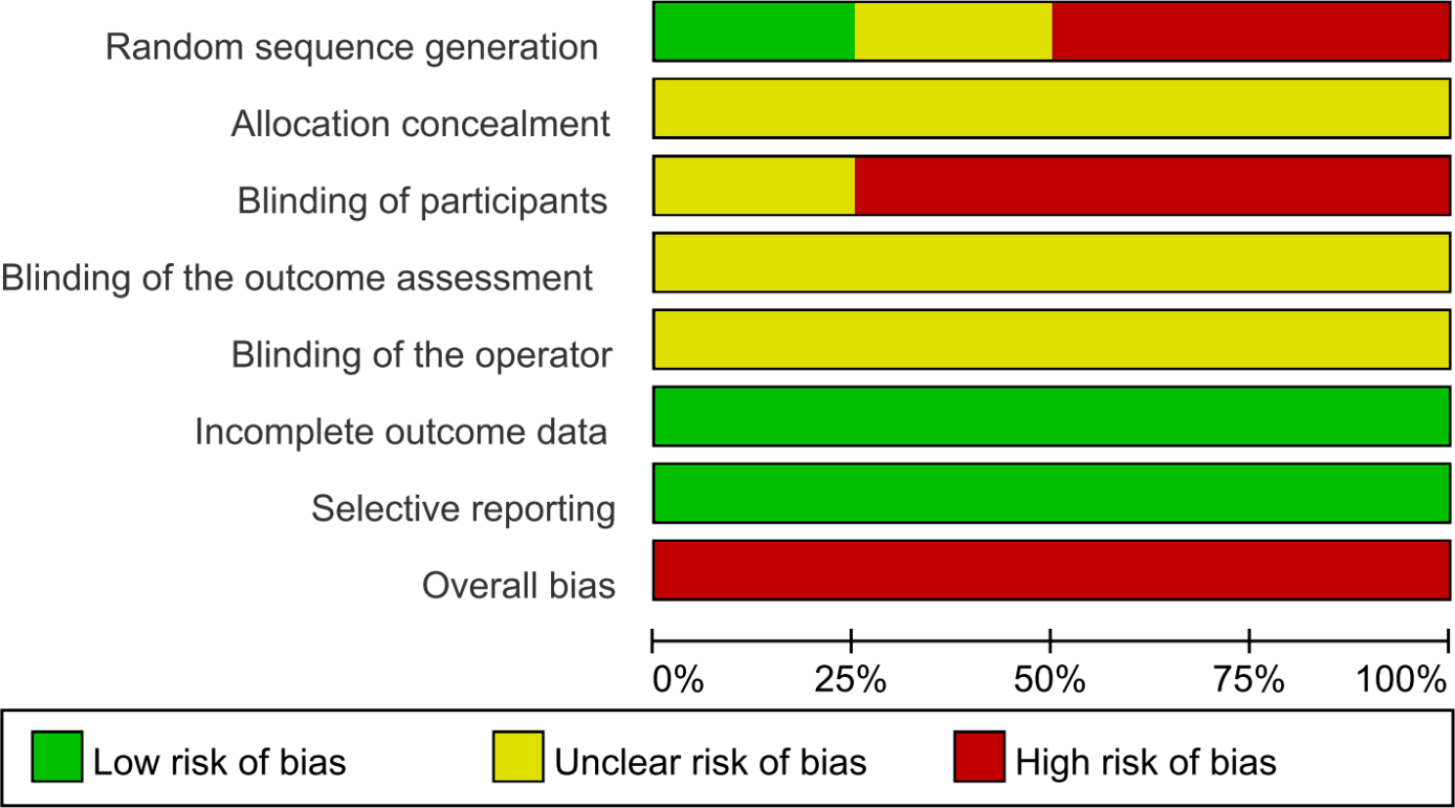
Risk of bias of experimental studies using Cochrane Collaboration’s tool

Table 5 presents a summary of the quality assessment of observational studies. Three publications were considered to be of good quality with low risk, providing adequate information about the population and objectives of the study, and how the outcomes and exposures were measured. 18 studies were rated as fair, while four publications were rated as poor. In terms of the experimental studies, two studies showed a high risk of random sequence generation since subjects were not randomly assigned to the experimental and control groups. Most studies also exhibited a high risk of bias in blinding of participants because participants were aware of their grouping under different intervention conditions. See Fig. 2 for a graphical representation of the risk of bias assessment.

**Table 5:** Quality assessment of included observational studies

Author(s)	Question number according to NIH Quality Assessment Tool													
	1	2	3	4	5	6	7	8	9	10	11	12	13	14
Almohamad et al. [24] 2022	Y	Y	N	Y	N	Y	NR	N	Y	N	Y	NR	NA	Y
Al-Zahrani et al. [25] 2005	Y	Y	N	Y	N	Y	Y	Y	Y	N	Y	NR	NA	Y
Al-Zahrani et al. [26] 2005	Y	Y	Y	Y	N	N	N	N	Y	N	NR	NR	NA	NR
Bawadi et al. [27] 2011	Y	N	Y	Y	Y	N	NR	N	Y	N	Y	NR	Y	Y
Bazyar et al. [28] 2019	Y	Y	NR	Y	N	N	Y	Y	Y	N	Y	NR		
Cueto et al. [29] 2005	Y	Y	NA	NA	Y	Y	NR	Y	NR	Y	NR	Y		
Han et al. [30] 2010	Y	Y	Y	Y	Y	Y	NR	Y	Y	N	Y	NR	NA	Y
Han et al. [31] 2016	Y	N	Y	Y	N	Y	N	N	NR	N	NR	NR	NA	Y
Han et al. [32] 2017	Y	N	Y	Y	N	Y	NR	Y	Y	N	Y	NR	NA	Y
Han et al. [33] 2018	Y	N	Y	Y	N	Y	NR	Y	Y	N	Y	NR	NA	Y
Han et al. [34] 2019	Y	N	Y	Y	N	N	N	Y	Y	N	Y	NR	Y	Y
Hasan et al. [35] 2021	Y	Y	Y	Y	Y	Y	N	Y	Y	N	Y	NR	Y	Y
Hoppe et al. [36] 2017	Y	Y	Y	Y	Y	NR	NR	Y	Y	N	Y	NR	NA	Y
Hwang et al. [37] 2022	Y	N	Y	Y	Y	N	Y	N	NR	N	NR	NR	Y	Y
Iwasaki et al. [38] 2023	Y	N	N	Y	Y	N	N	N	Y	N	Y	Y	NA	Y
Kongstad et al. [39] 2017	Y	Y	Y	Y	N	N	NR	Y	Y	N	Y	NR	NA	Y
Marruganti et al. [40] 2022	Y	Y	Y	Y	N	NR	N	N	Y	N	Y	NR	NA	Y
Marruganti et al. [41] 2023	Y	N	NR	Y	N	N	Y	Y	Y	N	Y	NR	NA	Y
Merchant et al. [43] 2003	Y	Y	Y	Y	N	Y	Y	Y	Y	Y	Y	NR	Y	Y
Munther et al. [44] 2019	Y	Y	NR	Y	Y	N	NR	N	NR	N	Y	NR	Y	NR
Sakki et al. [45] 1995	Y	N	Y	Y	N	Y	Y	N	Y	Y	Y	NR	NA	Y
Sanders et al. [46] 2009	Y	Y	N	Y	Y	Y	NR	N	Y	Y	NR	Y		
Samnieng et al. [47] 2013	Y	Y	N	Y	Y	N	NR	N	Y	N	Y	NR	Y	Y
Schmidt et al. [48] 2022	Y	Y	Y	Y	N	Y	NR	N	Y	N	Y	NR	Y	Y
Oliveira et al. [49] 2015	Y	N	Y	Y	N	Y	Y	Y	Y	Y	Y	NR	Y	Y
Yu et al. [52] 2011	Y	Y	Y	Y	N	N	NR	Y	NR	N	Y	NR	NA	Y

Y: Yes; N: No; NR: Not Reported; NA: Not Applicable

### Meta-analysis

**Fig. 3:**
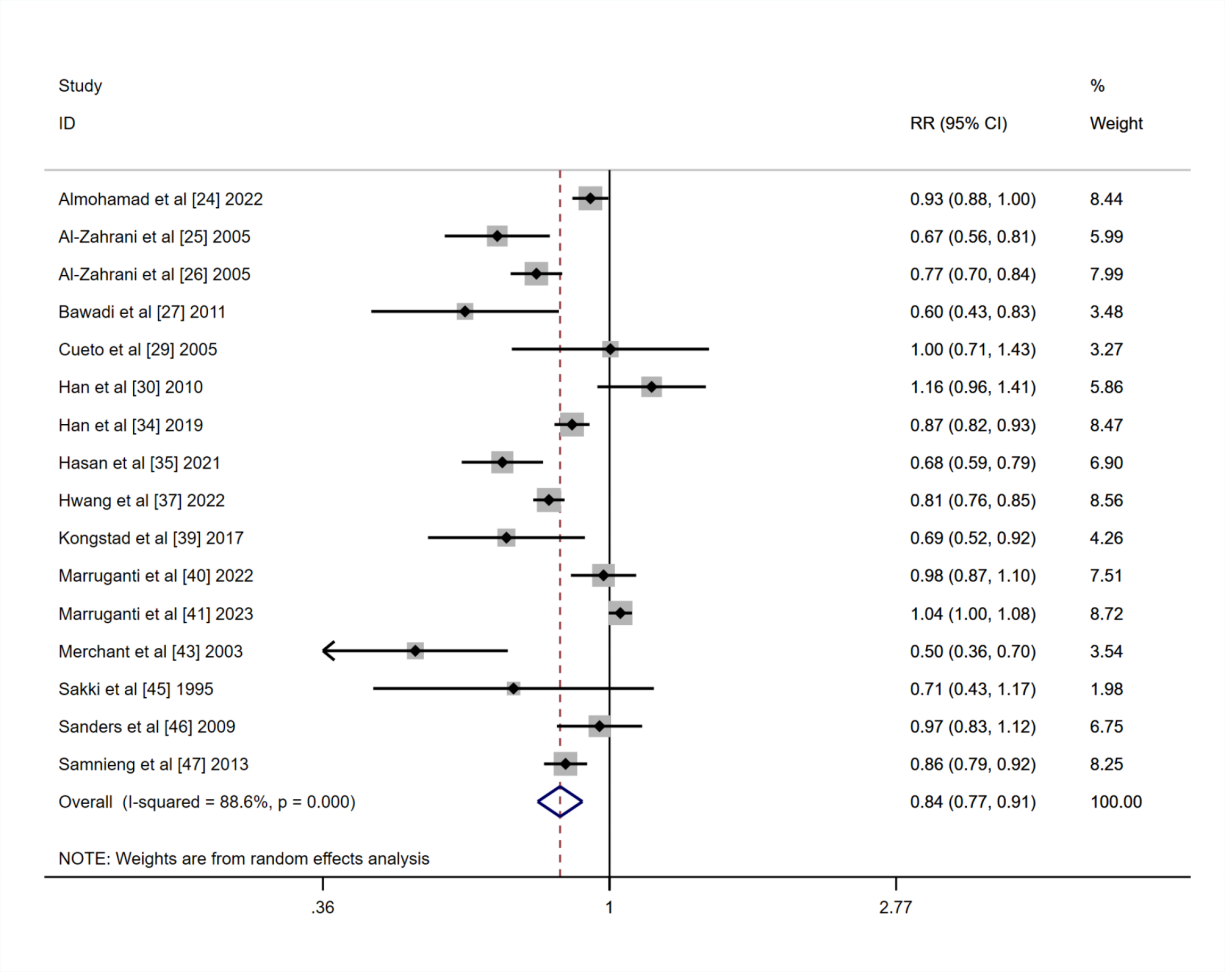
Forest plot of the relationship between exercise and prevalence of periodontitis

**Fig. 4:**
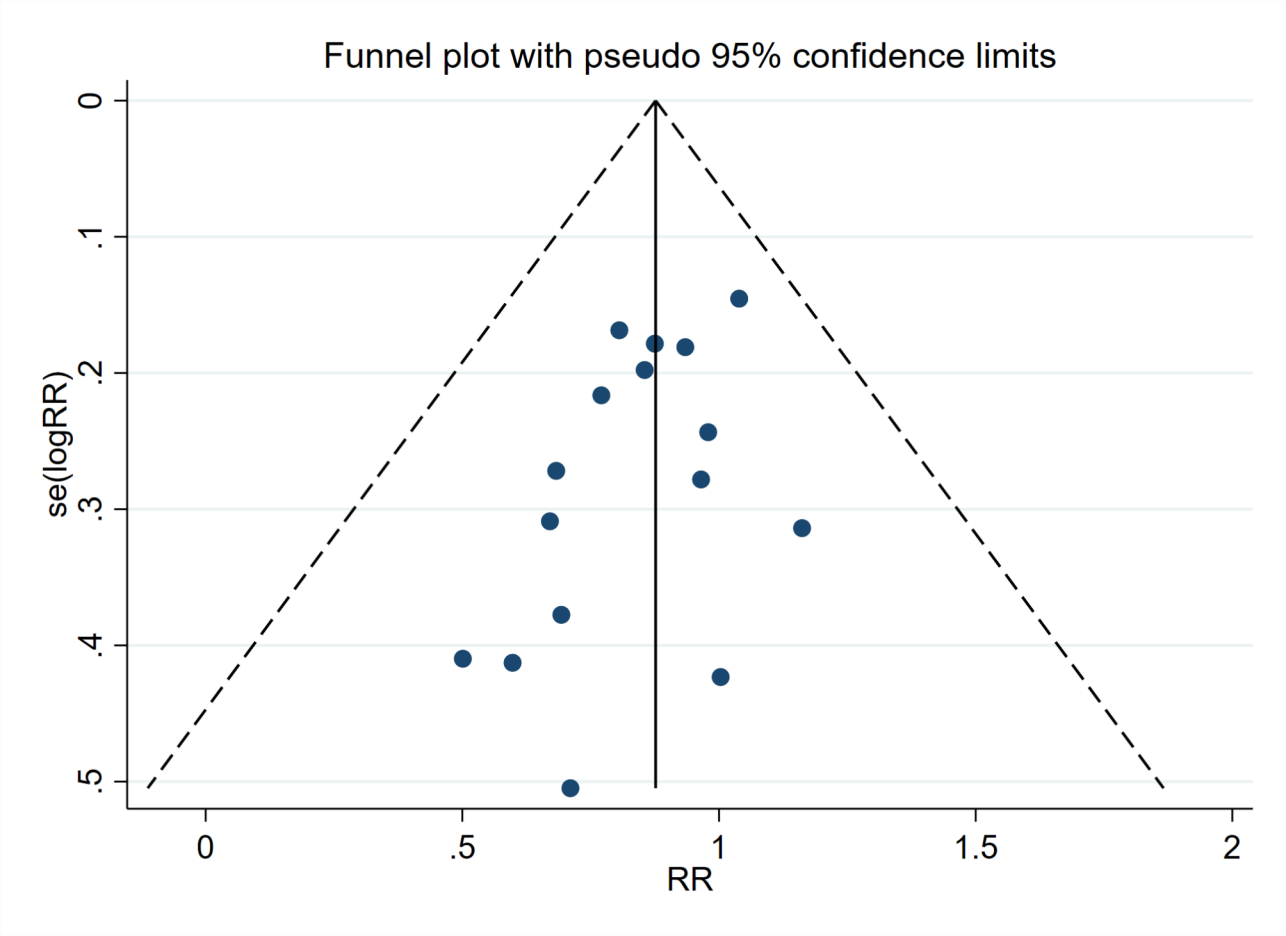
Funnel plot of the relationship between exercise and prevalence of periodontitis

Among 30 selected articles, 16 were included in the meta-analysis, while 14 publications were excluded due to variability in study outcomes and methodologies. The results presented high heterogeneity (I2 = 88) with statistical significance. Since the selected studies were not functionally equivalent, Random-effects models were used to generalize the results from meta-analysis. The prevalence of periodontitis was 27.85% in the active group and 33.88% in the inactive group, respectively. The Forest plot demonstrated a risk ratio of 0.84 (95%CI: 0.77, 0.91) between the active group and the inactive group (P < 0.01), which indicated a positive association between exercise and periodontitis (Fig. 3). The funnel plot did not reveal any publication bias (Fig. 4).

## Discussion

Regular exercise is considered an essential aspect of many individuals’ lives, and it has been postulated to positively impact periodontitis through various mechanisms, including improved insulin sensitivity, decreased incidence of lifestyle-related diseases and obesity, stress reduction, and decreased inflammation reactions [53]. Furthermore, lifestyle and obesity are closely linked to the development of periodontitis [54]. Despite this, few studies have investigated the relationship between exercise and periodontitis. Two previous published meta-analyses have tried to clarify the underlying relationship between exercise and periodontitis [17, 18]. However, the results should be considered carefully to some extent due to the limited research available and certain risk of bias. In addition, these previous meta-analyses only included observational studies. Conversely, after a comprehensive literature review, 30 studies were selected for inclusion in our study. Moreover, in this study, we included both experimental studies and observational studies. Following a systematic review, the included studies demonstrated a significant correlation between exercise and periodontitis. To our knowledge, this is one of the first reviews that focus on the influence of taking exercise on periodontitis.

As the fourth leading risk factor for global mortality, physical inactivity has been identified as a modifiable risk factor for various diseases, such as diabetes mellitus, hypertension, cardiovascular disease, and osteoporosis [55]. Exercise and physical activity have been shown to enhance life quality and increase life expectancy, resulting in an increasing focus on these activities by organizations [56]. The terms exercise and physical activity have been currently used interchangeably in the literature [57]. Therefore, we included both of them in the systematic review to improve the reliability. Previous research has indicated that exercise is linked to a favorable inflammatory profile, which may provide some protection against oral diseases [58]. The World Health Organization (WHO) recommends a moderate exercise pattern, defined as at least 75 min of running or 150 min of brisk walking per week, which has been associated with a lower prevalence of lifestyle-related diseases [3]. However, there are still a significant number of individuals who fail to meet these recommendations, which needs to be taken seriously.

Periodontitis refers to pathologic loss of alveolar bone and periodontal ligament, involving complex dynamic interactions among destructive immune responses and specific bacterial pathogens [59]. Our results showed that taking exercise is considered a health-promoting measure, which results in a decrease in the prevalence and severity of periodontitis. The underlying mechanism accounting for this reduction in prevalence may be associated with the influence of taking exercise on cytokine production and immune modulation [60, 61]. C-reactive protein (CRP), a by-product of vitamin K metabolism, is significantly correlated with periodontitis. Studies have revealed that physical exercise can modulate several cytokines, including CRP [13]. Thus, maintaining regular physical activity should be encouraged to improve periodontitis.

This study performed a literature review and meta-analysis, which confirmed the relationship between exercise and periodontitis. Among the 30 studies included, 20 reported a positive relationship between the level of exercise and periodontitis, the remaining 10 articles did not report significant differences. Notably, none of the included studies reported any negative effects regarding the influence of exercise on periodontitis. The meta-analysis revealed a risk ratio of 0.84 (95% CI: 0.77, 0.91) between the exercise group and the inactive group (P < 0.01), although there was considerable heterogeneity, which indicates that engaging in physical exercise is inversely associated with the presence and severity of periodontitis.

The selected studies proposed several influential factors that may impact periodontitis, including maintaining a healthy weight, consuming a nutritious diet, reducing sedentary behavior, and avoiding cigarette smoking. Among the studies, 7 evaluated diet quality and found a strong association between consuming a high-quality diet and improving periodontitis. In addition, 4 studies suggested that maintaining normal body weight is important in preventing periodontitis, as obesity-related inflammation can induce bacterial proliferation and produce inflammatory markers in fat tissues, which may exacerbate periodontitis. 3 studies analyzed the influence of tobacco smoking habits on periodontitis and concluded that cigarette smoking may affect the periodontal status through total salivary antioxidant activity. Furthermore, higher sedentary behavior was found to be associated with higher odds of periodontitis. Accordingly, in addition to emphasizing exercise, interventions should also focus on the factors mentioned above, including improving diet quality, maintaining normal body weight, reducing sedentary behavior, and avoiding cigarette smoking.

The present study has several limitations. Firstly, the measurement of exercise involved multiple tools, including different versions of questionnaires, strength, and maximal oxygen consumption, resulting in challenges in drawing definitive conclusions. Additionally, the included studies had varying sample sizes, with a significant gap between the smallest and largest numbers, and were mostly conducted in developed countries, which may have affected the quality of data analysis. Finally, most selected studies were observational and did not employ measures to reduce bias such as random allocation. Accordingly, in order to address existing gaps in evidence, future researches in larger population samples with a longer follow-up time are needed to understand the real role of exercise on the prevalence of periodontitis. Moreover, more intervention researches are needed to establish a possible cause-effect association between exercise and the management of periodontitis.

## Conclusion

Despite current knowledge gaps, the present review and meta-analysis systematically summarized current epidemiological data, providing evidence of a significant correlation between exercise and a lower occurrence of periodontitis. Therefore, taking exercise is proposed as a critical component of periodontitis management. Future researches in larger population samples with a longer follow-up time are needed to understand the real role of exercise on the prevalence of periodontitis. Moreover, more intervention researches are needed to establish a possible cause-effect association between exercise and the management of periodontitis, to help dental health providers take measures in clinical scenarios.

## Data Availability

The datasets generated during and/or analyzed during the current study are available from the corresponding author on reasonable request.

## References

[1] Chodzko-Zajko WJ, Proctor DN, American College of Sports Medicine (2009). American College of Sports Medicine position stand. Exercise and physical activity for older adults. Med Sci Sports Exerc.

[2] Williams PT, Franklin B (2007). Vigorous exercise and diabetic, hypertensive, and hypercholesterolemia medication use. Med Sci Sports Exerc.

[3] Minich DM, Bland JS (2013). Personalized lifestyle medicine: relevance for nutrition and lifestyle recommendations. ScientificWorldJournal.

[4] Mehta KM, Yaffe K, Brenes GA (2007). Anxiety symptoms and decline in physical function over 5 years in the health, aging and body composition study. J Am Geriatr Soc.

[5] Pratt M, Macera CA, Wang G (2000). Higher direct medical costs associated with Physical Inactivity. Phys Sportsmed.

[6] Mariotti A, Hefti AF. Defining periodontal health. BMC Oral Health. 2015;15 Suppl 1(Suppl 1):S6. 10.1186/1472-6831-15-S1-S6 10.1186/1472-6831-15-S1-S6 PMC4580771 26390888

[7] Papapanou PN, Sanz M, Buduneli N, Dietrich T, Feres M, Fine DH, Flemmig TF, Garcia R, Giannobile WV, Graziani F et al. Periodontitis: Consensus report of workgroup 2 of the 2017 World Workshop on the Classification of Periodontal and Peri-Implant Diseases and Conditions. J Clin Periodontol. 2018;45 Suppl 20:S162-S170. 10.1111/jcpe.12946 10.1111/jcpe.12946 29926490

[8] Nazir MA (2017). Prevalence of periodontal Disease, its association with systemic Diseases and prevention. Int J Health Sci (Qassim).

[9] Tonetti MS, Van Dyke TE, working group 1 of the joint EFP/AAP workshop (2013). Periodontitis and atherosclerotic Cardiovascular Disease: consensus report of the Joint EFP/AAP Workshop on Periodontitis and systemic Diseases. J Periodontol.

[10] Tar I, Csősz É, Végh E (2021). Salivary citrullinated proteins in rheumatoid arthritis and associated periodontal Disease. Sci Rep.

[11] Kassebaum NJ, Bernabé E, Dahiya M (2014). Global burden of severe periodontitis in 1990–2010: a systematic review and meta-regression. J Dent Res.

[12] Ramseier CA, Woelber JP, Kitzmann J (2020). Impact of risk factor control interventions for smoking cessation and promotion of healthy lifestyles in patients with periodontitis: a systematic review. J Clin Periodontol.

[13] Lieske B, Moszka N, Borof K (2023). Association between an anti-inflammatory dietary score and periodontitis-evidence from the Population-based Hamburg City Health Study. Nutrients.

[14] Amaranath BJ, Gupta S, Kumar S (2023). Assessment of Periodontal Health Status among the Male Adult Population with a dual habit of Smoking and Gutkha Chewing: a cross-sectional study. J Pharm Bioallied Sci.

[15] Stewart LK, Flynn MG, Campbell WW (2007). The influence of exercise training on inflammatory cytokines and C-reactive protein. Med Sci Sports Exerc.

[16] Hammonds TL, Gathright EC, Goldstein CM (2016). Effects of exercise on c-reactive protein in healthy patients and in patients with Heart Disease: a meta-analysis. Heart Lung.

[17] Rodríguez-Archilla A, Padrón-Curiel DA (2022). Influence of physical exercise on periodontal Disease: a meta-analysis. Int J Dent Sci.

[18] Ferreira RO, Corrêa MG, Magno MB (2019). Physical activity reduces the prevalence of Periodontal Disease: systematic review and Meta-analysis. Front Physiol.

[19] Page MJ, McKenzie JE, Bossuyt PM (2021). The PRISMA 2020 statement: an updated guideline for reporting systematic reviews. BMJ.

[20] Schardt C, Adams MB, Owens T (2007). Utilization of the PICO framework to improve searching PubMed for clinical questions. BMC Med Inform Decis Mak.

[21] Cumpston M, Li T, Page MJ (2019). Updated guidance for trusted systematic reviews: a new edition of the Cochrane Handbook for Systematic Reviews of Interventions. Cochrane Database Syst Rev.

[22] Borenstein M, Hedges LV, Higgins JP, Rothstein HR (2010). A basic introduction to fixed-effect and random-effects models for meta-analysis. Res Synth Methods.

[23] Alkan B, Guzeldemir-Akcakanat E, Odabas-Ozgur B (2020). Effects of exercise on periodontal parameters in obese women. Niger J Clin Pract.

[24] Almohamad M, Krall Kaye E, Mofleh D (2022). The association of sedentary behaviour and physical activity with periodontal Disease in NHANES 2011–2012. J Clin Periodontol.

[25] Al-Zahrani MS, Borawski EA, Bissada NF (2005). Increased physical activity reduces prevalence of periodontitis. J Dent.

[26] Al-Zahrani MS, Borawski EA, Bissada NF (2005). Periodontitis and three health-enhancing behaviors: maintaining normal weight, engaging in recommended level of exercise, and consuming a high-quality diet. J Periodontol.

[27] Bawadi HA, Khader YS, Haroun TF (2011). The association between periodontal Disease, physical activity and healthy diet among adults in Jordan. J Periodontal Res.

[28] Bazyar H, Adibmanesh A, Javid AZ (2019). The relationship between metabolic factors and anthropometric indices with periodontal status in type 2 Diabetes Mellitus patients with chronic periodontitis. Obes Med.

[29] Cueto A, Mesa F, Bravo M (2005). Periodontitis as risk factor for acute Myocardial Infarction. A case control study of Spanish adults. J Periodontal Res.

[30] Han DH, Lim SY, Sun BC (2010). The association of metabolic syndrome with periodontal Disease is confounded by age and Smoking in a Korean population: the Shiwha-Banwol Environmental Health Study. J Clin Periodontol.

[31] Han K, Hwang E, Park JB (2016). Excessive consumption of Green Tea as a risk factor for Periodontal Disease among Korean adults. Nutrients.

[32] Han K, Park JB (2017). Association between oral health behavior and periodontal Disease among Korean adults: the Korea national health and nutrition examination survey. Med (Baltim).

[33] Han K, Park YM, Park JB (2018). Evaluation of an association between long sleep duration and periodontal Disease among men and women using nationally representative data. Gac Sanit.

[34] Han SJ, Bae KH, Lee HJ (2019). Association between regular walking and periodontitis according to socioeconomic status: a cross-sectional study. Sci Rep.

[35] Hasan SMM, Rahman M, Nakamura K (2021). Relationship between Diabetes self-care practices and control of periodontal Disease among type2 Diabetes patients in Bangladesh. PLoS ONE.

[36] Hoppe CB, Oliveira JAP, Grecca FS (2017). Association between chronic oral inflammatory burden and physical fitness in males: a cross-sectional observational study. Int Endod J.

[37] Hwang SY, Jang JH, Park JE (2022). Association between Healthy Lifestyle (Diet Quality, physical activity, normal body weight) and Periodontal Diseases in Korean adults. Int J Environ Res Public Health.

[38] Iwasaki M, Yoshihara A, Suwama K (2023). A cross-sectional study of the association between periodontitis and physical activity in the Japanese population. J Periodontal Res.

[39] Kongstad J, Enevold C, Christensen LB (2017). Impact of Periodontitis Case Criteria: a cross-sectional study of Lifestyle. J Periodontol.

[40] Marruganti C, Traversi J, Gaeta C (2022). Adherence to Mediterranean diet, physical activity level, and severity of periodontitis: results from a university-based cross-sectional study. J Periodontol.

[41] Marruganti C, Baima G, Grandini S (2023). Leisure-time and occupational physical activity demonstrate divergent associations with periodontitis: a population-based study. J Clin Periodontol.

[42] Mendoza-Núñez VM, Hernández-Monjaraz B, Santiago-Osorio E (2014). Tai Chi exercise increases SOD activity and total antioxidant status in saliva and is linked to an improvement of periodontal Disease in the elderly. Oxid Med Cell Longev.

[43] Merchant AT, Pitiphat W, Rimm EB (2003). Increased physical activity decreases periodontitis risk in men. Eur J Epidemiol.

[44] Munther S (2019). The effects of cigarette Smoking and exercise on total salivary antioxidant activity. Saudi Dent J.

[45] Sakki TK, Knuuttila ML, Vimpari SS (1995). Association of lifestyle with periodontal health. Community Dent Oral Epidemiol.

[46] Sanders AE, Slade GD, Fitzsimmons TR (2009). Physical activity, inflammatory biomarkers in gingival crevicular fluid and periodontitis. J Clin Periodontol.

[47] Samnieng P, Ueno M, Zaitsu T (2013). The relationship between seven health practices and oral health status in community-dwelling elderly Thai. Gerodontology.

[48] Schmidt J, Vogel M, Poulain T (2022). Association of Oral Health Conditions in adolescents with social factors and obesity. Int J Environ Res Public Health.

[49] Oliveira JA, Hoppe CB, Gomes MS (2015). Periodontal Disease as a risk indicator for poor physical fitness: a cross-sectional observational study. J Periodontol.

[50] Omori S, Uchida F, Oh S (2018). Exercise habituation is effective for improvement of periodontal Disease status: a prospective intervention study. Ther Clin Risk Manag.

[51] Wernicke K, Grischke J, Stiesch M (2021). Influence of physical activity on periodontal health in patients with type 2 Diabetes Mellitus. A blinded, randomized, controlled trial. Clin Oral Investig.

[52] Yu YH, Lai YL, Cheung WS (2011). Oral health status and self-reported functional dependence in community-dwelling older adults. J Am Geriatr Soc.

[53] Pischon N, Heng N, Bernimoulin JP (2007). Obesity, inflammation, and periodontal Disease. J Dent Res.

[54] Jepsen S, Suvan J, Deschner J (2020). The association of periodontal Diseases with metabolic syndrome and obesity. Periodontol 2000.

[55] Banting LK, Dimmock JA, Grove JR (2011). The impact of automatically activated motivation on exercise-related outcomes. J Sport Exerc Psychol.

[56] Peralta LR, Cinelli RL, Cotton W (2022). The barriers to and facilitators of Physical Activity and Sport for Oceania with Non-European, non-asian (ONENA) Ancestry Children and adolescents: a mixed studies systematic review. Int J Environ Res Public Health.

[57] Speck BJ (2002). From exercise to physical activity. Holist Nurs Pract.

[58] Mathur N, Pedersen BK (2008). Exercise as a mean to control low-grade systemic inflammation. Mediators Inflamm.

[59] Uchida F, Oh S, Shida T (2021). Effects of Exercise on the oral microbiota and saliva of patients with non-alcoholic fatty Liver Disease. Int J Environ Res Public Health.

[60] Codella R, Della Guardia L, Terruzzi I (2021). Physical activity as a proxy to ameliorate inflammation in patients with type 2 Diabetes and periodontal Disease at high cardiovascular risk. Nutr Metab Cardiovasc Dis.

[61] Slots J (2017). Periodontitis: facts, fallacies and the future. Periodontol 2000.

